# Observation of the non-linear Meissner effect

**DOI:** 10.1038/s41467-022-28790-y

**Published:** 2022-03-07

**Authors:** J. A. Wilcox, M. J. Grant, L. Malone, C. Putzke, D. Kaczorowski, T. Wolf, F. Hardy, C. Meingast, J. G. Analytis, J.-H. Chu, I. R. Fisher, A. Carrington

**Affiliations:** 1 H. H. Wills Physics Laboratory, University of Bristol, Tyndall Avenue, Bristol, BS8 1TL UK; 2 Institute of Low Temperature and Structure Research, Polish Academy of Sciences, 50-950 Wroclaw, Poland; 3 Institute for Quantum Materials and Technologies, Karlsruhe Institute of Technology, 76021 Karlsruhe, Germany; 4 Geballe Laboratory for Advanced Materials and Department of Applied Physics, Stanford University, Stanford, CA 94305-4045 USA; 5 Stanford Institute for Materials and Energy Sciences, SLAC National Accelerator Laboratory, 2575 Sand Hill Road, Menlo Park, CA 94025 USA; 6 Present Address: Laboratory of Quantum Materials, Institute of Materials, École Polytechnique Fédérale de Lausanne (EPFL), 1015 Lausanne, Switzerland; 7 Present Address: Department of Physics, University of California, Berkeley, CA 94720 USA; 8 Present Address: Department of Physics, University of Washington, Seattle, WA 98195 USA

**Keywords:** Electronic properties and materials, Superconducting properties and materials

## Abstract

A long-standing theoretical prediction is that in clean, nodal unconventional superconductors the magnetic penetration depth *λ*, at zero temperature, varies linearly with magnetic field. This non-linear Meissner effect is an equally important manifestation of the nodal state as the well studied linear-in-*T* dependence of *λ*, but has never been convincingly experimentally observed. Here we present measurements of the nodal superconductors CeCoIn_5_ and LaFePO which clearly show this non-linear Meissner effect. We further show how the effect of a small dc magnetic field on *λ*(*T*) can be used to distinguish gap nodes from non-nodal deep gap minima. Our measurements of KFe_2_As_2_ suggest that this material has such a non-nodal state.

## Abstract (web summary)

The non-linear Meissner effect is a key manifestation of unconventional nodal superconductors but its experimental evidence has been elusive. Here, the authors observe the nonlinear Meissner effect in nodal superconductors CeCoIn_5_ and LaFePO.

## Introduction

Determination of the symmetry and momentum dependent structure of the superconducting energy gap Δ(***k***) is of fundamental importance as this provides a strong guide to microscopic theories of superconductivity^1^. Measurements of the temperature dependence of the magnetic penetration depth *λ*(*T*) have proved extremely useful as *λ*(*T*) is directly related to the energy dependence of the quasiparticle density of states *N*(*E*) which in turn is related to Δ(***k***)^2^. For example, a line node in Δ(***k***) on a quasi-two-dimensional Fermi surface causes *N*(*E*) to vary linearly with energy and *λ* to vary linearly with *T*. In contrast, if Δ(***k***) is finite for all ***k*** then *λ*(*T*) will vary exponentially for *T* much less than the minimum gap^3^.

It was theoretically proposed^4^ that the magnetic field dependence of *λ* also depends strongly on Δ(***k***) and thus provides an alternative test of the pairing state. In a nodal superconductor in the clean limit, the theory for this non-linear Meissner effect predicts that *λ* is a linear function of *H* at *T* = 0, with a field-scale *H*_0_ which is of order the thermodynamic critical field *H*_*c*_ (i.e., Δ*λ*(*H*)/*λ*_0_ = *H*/*H*_0_). However, despite the considerable experimental effort, this long-standing prediction has not been convincingly observed experimentally. Although the change in *λ* with the field, Δ*λ*(*H*), was found to be approximately linear in the cuprate superconductor YBa_2_Cu_3_O_6+*x*_ (Y123)^5,6^, Δ*λ*(*H*) had a very weak temperature and angle dependence. The latter might be explained by the orthorhombicity of Y123^7^ but the lack of temperature dependence of Δ*λ*(*H*) is in serious disagreement with theory and strongly suggests that the observed effects in Y123 were of extrinsic origin^5,6^. Attempts to observe the effect in Y123 through transverse torque measurements were also unsuccessful^8^. Later it was predicted^9^ that the non-linear current relation which leads to the linear *λ*(*H*) in nodal superconductors should also give rise to intermodulation distortion (IMD) in the microwave response. Although extrinsic defects also give rise to IMD, the theoretically predicted increase in the amplitude of the IMD as the temperature is decreased was observed in high-quality films of Y123^10^ suggesting that the intrinsic response is present and adds to the extrinsic IMD. Despite the IMD results, the lack of experimental evidence for the predicted response of *λ* with field remains an outstanding issue which could suggest a problem with the standard quasiclassical theory of unconventional superconductivity.

Here we present measurements of *λ*(*H*) for two putative nodal superconductors, CeCoIn_5_ and LaFePO, which show convincingly that the predicted field dependence of *λ* is present in nodal superconductors and this field dependence decreases with increasing temperature as expected. Our measurements of a third material, KFe_2_As, are markedly different which we argue results from a small but finite non-nodal gap in this superconductor. We show how the combination of the effect of field and temperature on *λ* can be used to distinguish between the case where there is a small density of impurities in a nodal superconductor from the case where there is a small but finite energy gap.

CeCoIn_5_ has been extensively studied using many probes including specific heat, thermal conductivity *κ*(*T*)^11,12^ and *λ*(*T*)^13–17^ with the results consistent with *d*-wave superconductivity with line nodes. *λ*(*T*) data showed some small differences from the clean-limit *d*-wave form which was interpreted as evidence of proximity to a quantum critical point^16–18^, or the effect of non-local electrodynamics near the node^14^. For LaFePO, *λ*(*T*)^19,20^ and *κ*(*T*)^21,22^ measurements indicate line nodes but with theory suggesting Δ(***k***) to have *A*_2*g*_ (*s*-wave) symmetry^23^. KFe_2_As_2_ is more controversial; although *λ*(*T*) has a strong, quasi-linear dependence consistent with gap nodes^24^, specific heat measurements^25^ show evidence for very small gaps which may be difficult to distinguish from true nodes. *κ*(*T*) measurements have been argued to show universal behaviour consistent with *d*-wave order^26^, although this has been disputed^27^. Additionally, angle-resolved photoemission measurements have indicated the presence of eight-fold nodes on one of the Fermi surface sheets^28^. This result should be viewed with some caution since the measurement was carried out at *T* = 1.5 K, and given *T*_*c*_ = 3.4 K, the gap structure will not have fully developed to its zero temperature value. Studies of *λ*(*T*) and the effect of electron irradiation on the doped series Ba_1−*x*_K_*x*_Fe_2_As_2_, in combination with theoretical work, suggest that Ba_1−*x*_K_*x*_Fe_2_As_2_ has a highly anisotropic gap which may change sign on some parts of the Fermi surface as *x* → 1^29^. Hence it remains an open question whether KFe_2_As_2_ has gap nodes or not.

## Results

### Temperature dependence of the zero-field penetration depth

**Fig. 1:**
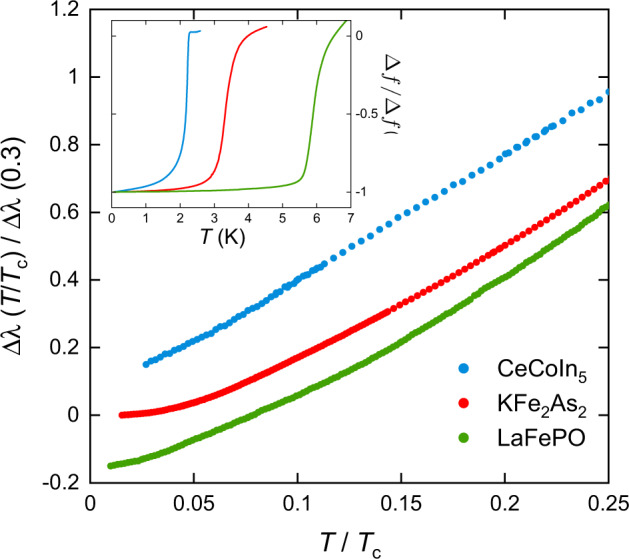
Temperature dependence of in-plane *λ* relative to its value at the lowest temperature Δ*λ*(*T*) for CeCoIn_5_, LaFePO, and KFe_2_As_2_. For each material, Δ*λ*(*T*) is normalised by its value at *T*/*T*_*c*_ = 0.3. The data for CeCoIn_5_ and LaFePO have been shifted by ±0.15 along the *λ* axis for clarity. The inset shows the RF oscillator frequency change over the full temperature range to emphasise the superconducting transitions. Here Δ*f* is measured relative to *f*(*T*) just above *T*_*c*_ and normalised to −1 at the lowest temperature. The mid-points of the transition are 2.1, 3.2 and 5.9 K for CeCoIn_5_, KFe_2_As_2_ and LaFePO respectively. For KFe_2_As_2_ and CeCoIn_5_ the RF field is parallel to the *ab* plane, and for LaFePO the RF field is parallel to the *c*-axis. Note that these transitions appear broader than the true distribution of *T*_*c*_ in the sample because of the strong *T* dependence of the normal state skin-depth and the finite sample thickness.

The temperature dependence of the in-plane *λ* for single crystal samples of CeCoIn_5_, LaFePO, and KFe_2_As_2_, in zero applied dc field and negligibly small ac probe field, is shown in Fig. 1. The data for all three materials are similar to previous reports and show a predominately linear temperature dependence of *λ* for *T* ≪ *T*_*c*_ as expected for a superconductor with gap nodes, or deep gap minima, in the presence of a low density of impurities^30^. The linear behaviour of *λ*(*T*) attests to the high quality of our samples showing that they have a very low pair-breaking disorder.

For CeCoIn_5_ and KFe_2_As_2_ the measurements were performed with *H* parallel to the *ab*-plane of the material to minimise demagnetising effects and potential non-local effects (Supplementary Note 3). As the samples are thin and neither material is very strongly anisotropic, the measured Δ*λ*(*T*) in this geometry is predominately the in-plane response but will also contain a small contribution from the out-of-plane *λ*(*T*) (Supplementary Note 3). For LaFePO, we used the *H*∥*c* geometry because of the larger anisotropy of *λ*(*T*) and the thicker *c*-axis dimension of the sample.

### Magnetic field dependence of the penetration depth

**Fig. 2:**
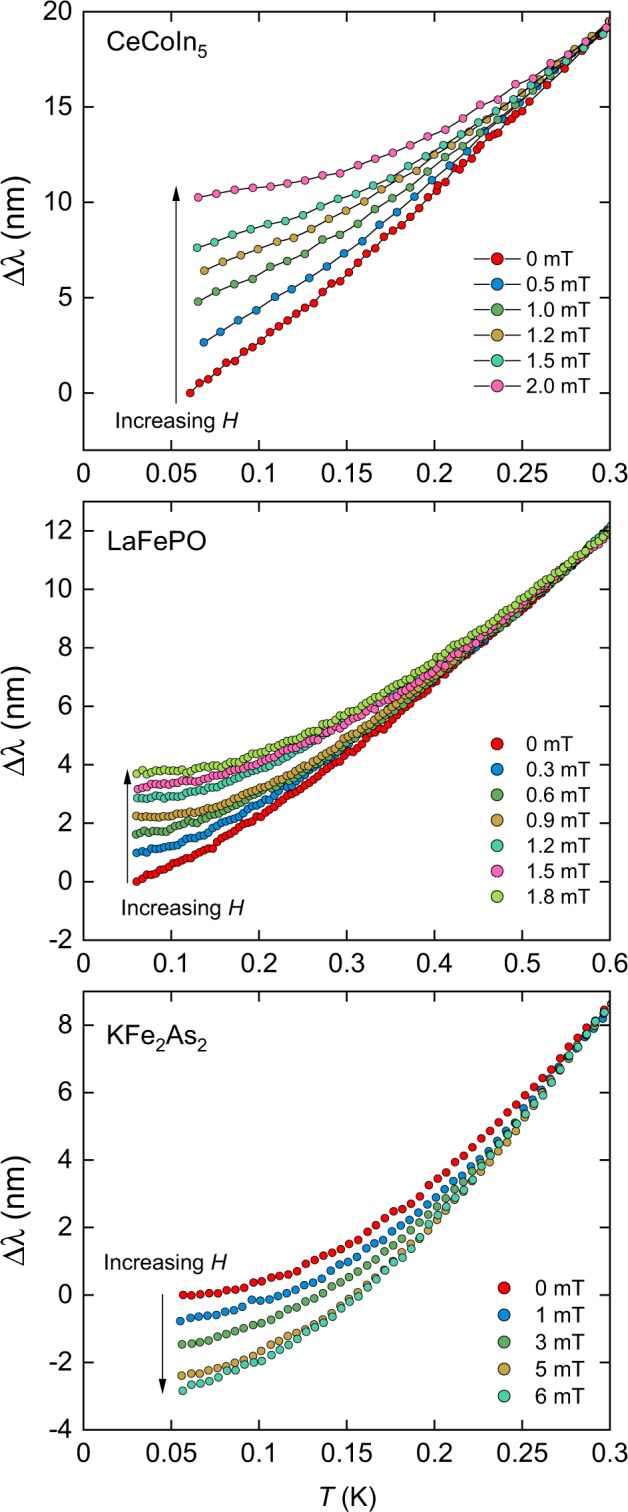
Effect of magnetic field on the temperature dependence of Δ*λ*. The finite field data have been shifted along the *λ* axis to coincide with the zero-field result at *T* = 0.3 K for CeCoIn_5_ and KFe_2_As_2_ and *T* = 0.6 K for LaFePO, in order to emphasise the progressive change in the temperature dependence of *λ* with the field as in Fig. 4.

In Fig. 2, we show the effect of a small dc field on *λ*(*T*) for all three materials. Here we have normalised the results in finite field to coincide with the zero-field data at the highest temperature in the figure to highlight the systematic changes in *λ*(*T*) with the field, and remove any field-dependent background effects (see Methods). For both CeCoIn_5_ and LaFePO, *λ*(*T*) becomes less temperature-dependent with the increasing field so that the normalised Δ*λ*(*T*) increases with the field at the lowest temperature. For KFe_2_As_2_, the effect of *H* is the opposite, with the temperature dependence of *λ*(*T*) becoming stronger so that Δ*λ* decreases with *H* at the lowest temperature. Note this decrease is an artefact of the high-temperature normalisation. We expect the absolute values of *λ* to always increase with *H* in all cases. The materials remain in the Meissner state for all *T* shown in this figure as *H* < *H*_*p*_(*T*) (the field of first flux penetration, which was measured by dc magnetisation for each sample, see Supplementary Note 4). For LaFePO, measured in the *H*∥*c* geometry, the surface fields will be non-uniform because of demagnetising effects, however, the effect of this on the measured surface-averaged *λ*(*H*) is estimated to be small (see Supplementary Note 9).

**Fig. 3:**
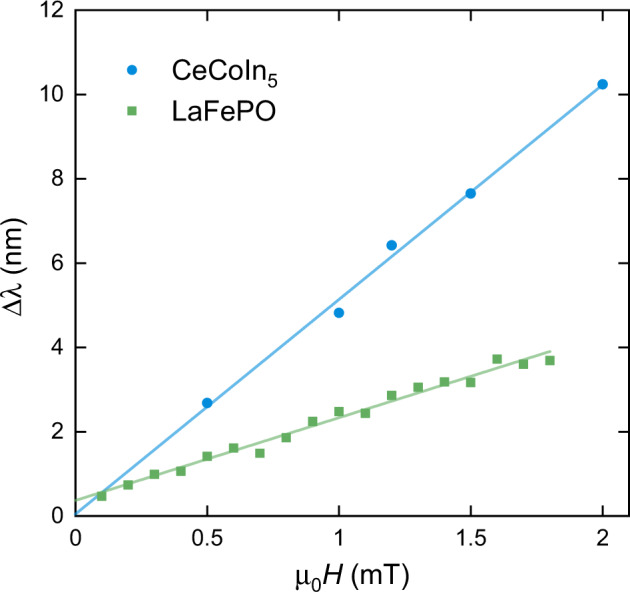
Field dependence of Δ*λ* at the lowest temperatures (65 mK) relative to the change at *T* = 0.3 K (CeCoIn_5_) or *T* = 0.6 K (LaFePO). The lines are linear fits to the data. Note that no correction for demagnetising effects has been made.

**Fig. 4:**
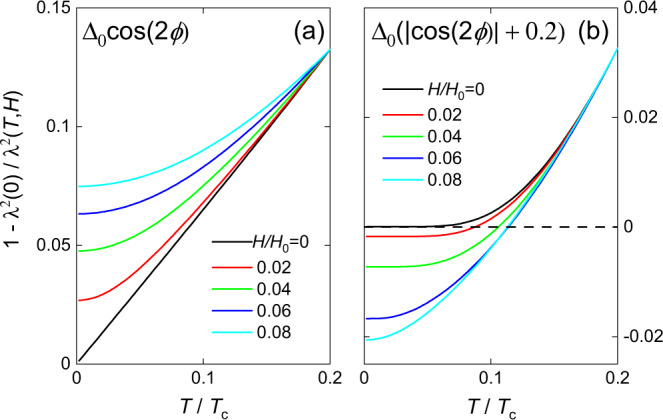
Calculated temperature dependence of the normalised superfluid density *λ*^2^(0)/*λ*^2^(*T*, *H*). **a** *d*-wave gap structure. **b** Gap structure is strongly anisotropic but with a small, finite gap. Both cases are in the clean limit and the finite field results have been shifted vertically so that they coincide with the *H* = 0 results at *T*/*T*_*c*_ = 0.2 for comparison with our experimental results. Unshifted results and details of the calculations are given in the SI. Note: $1-{\lambda }_{0}^{2}/{\lambda }^{2}(T,H)\simeq 2{{\Delta }}\lambda (T,H)/{\lambda }_{0}$ for small Δ*λ*(*T*, *H*).

In Fig. 3, we show that for both CeCoIn_5_ and LaFePO, the change in *λ* with *H* at the base temperature relative to the change at our reference temperature varies linearly with *H*. As Δ*λ*(*H*) decreases strongly with temperature (Fig. 2), normalising at this higher reference temperature can be expected to have only a small impact on Δ*λ*(*H*) at the lowest temperatures (see Supplementary Note 6). The observed linear-in-*H* behaviour of Δ*λ*(*H*) is in excellent agreement with the theory for the response of a clean, local, nodal superconductor—thus confirming the key prediction of ref. ^4^. A direct comparison with the theory can be made by comparing the temperature and field-dependent results in Fig. 2 to theoretical calculations in Fig. 4, where the results have been normalised in the same way. The magnitude of Δ*λ*(*H*) is in broad agreement with estimates (see Supplementary Note 10) using the material parameters, although a more quantitative analysis requires more detailed theoretical modelling, including multiband effects.

## Discussion

A problem with interpreting zero-field *λ*(*T*) to determine Δ(***k***) is the effect of impurities. Non-magnetic impurities produce gapless excitations for a range of ***k*** around a node and *λ*(*T*) ~ *T*^2^ below a characteristic temperature, T^*^, which depends on the impurity density^30^. Paramagnetic impurities also cause complications as these can cause upturns or flattening of *λ*(*T*) at low temperature^31^. In practice, it can be difficult to distinguish the case where there is a small density of impurities in a nodal superconductor from the case where there is a small but finite energy gap and so the results in Fig. 1 on their own may not provide decisive evidence for a nodal or non-nodal gap. This distinction is important because a node is strong evidence that Δ(***k***) changes sign on one or more Fermi surface sheets, which can prove to be strong evidence used to differentiate between different microscopic models of superconductivity.

The field dependence of *λ*(*T*) provides a route to distinguish between the nodal and non-nodal cases that is less sensitive to impurities than the zero-field temperature dependence of *λ* alone. The non-linear Meissner effect arises from the Doppler shift of the quasiparticle energies in a field, *δε* ∝ ***v***_***s***_ ⋅ ***v***_***F***_, where ***v***_***s***_ is the superflow velocity of the screening currents and ***v***_***F***_ is the Fermi velocity. Close to a node in Δ(***k***) this shifts the quasiparticle states below the Fermi level and hence they become occupied, producing backflow jets which reduce the effective superfluid density. At finite temperature, the field dependence of *λ* is reduced for *H* < H^*^, where H^*^ ~ *H*_0_*T*/*T*_*c*_, so that at fixed *H*, the temperature dependence of *λ* becomes weaker at low *T* (see Fig. 4a). In the case where the gap is isotropic, at low temperatures and small fields, the shift in the energy of the quasiparticle states is small compared to the gap for all ***k*** and so the effect is essentially absent^32^. However, when the gap is anisotropic but non-nodal, then close to the momentum where Δ(***k***) is minimum, this shift will move the minimum closer to zero, making the gap smaller, and hence, at sufficiently low temperature and for weak fields, Δ*λ*(*T*) will have a stronger *T*-dependence compared to zero field (see Fig. 4b). This distinct, contrasting change in the temperature dependence of *λ* with field is a key distinguishing factor between the nodal and non-nodal gap structures. Importantly, this difference is robust in the presence of a low density of impurities, both magnetic and non-magnetic (see below).

The markedly different behaviour of KFe_2_As_2_ compared to the other two materials, shown in Fig. 2, can be understood if Δ*λ*(*H*) is dominated by a small but finite energy gap, such as that modelled in Fig. 4b. In a multiband material like KFe_2_As_2_, there may be both nodal and small-yet-finite gapped sheets of Fermi surface^25^, and thus the measured *λ*(*T*, *H*) would be the sum of the contributions from all sheets. It is possible, therefore, that there could be a small contribution from a nodal sheet with sufficient impurity scattering to reduce its contribution to *λ*(*H*). However, our results show that the non-linear response is dominated by one or more sheets with a small but finite gap, which rules out there being nodes on all sheets. Further modelling could consider how impurities, causing inter- and intra-band scattering, would change the gap structure and hence the non-linear response.

An alternative way to analyse the data is to focus directly on the temperature dependence of *λ* rather than its field-dependent changes at a fixed temperature relative to that at some higher *T* reference point (as we have done in Figs. 2 and 3). In order to do this, we fit a power law to the normalised superfluid density; $\tilde{\rho }\equiv {\lambda }_{0}^{2}/{[{\lambda }_{0}+{{\Delta }}\lambda (T)]}^{2}=1-A{T}^{n}$ at low temperature. We fit $\tilde{\rho }$ rather than Δ*λ* because in the nodal case the former remains closer to the asymptotic low-temperature behaviour over a much larger range of temperature^5^. The resulting exponent *n* is only weakly dependent on the assumed value of *λ*_0_^19^. The fits were performed over the temperature range from base temperature up to 300 mK for CeCoIn_5_ and KFe_2_As_2_, and up to 400 mK for LaFePO. Note that this power-law behaviour is only an approximation to the true exponential behaviour at low temperature and zero field for the case of a finite gap, but is frequently used to fit experimental data^29^. The extracted exponent also depends slightly on the temperature range of the fit even for the nodal case, as a true power-law is theoretically only found in the asymptotic low-*T* behaviour (see Supplementary Note 7 for how *n* depends on the temperature range of the fit). For KFe_2_As_2_, *n*(*H* = 0) appears to be higher than what is reported in ref. ^29^ (*n* ≃ 1.45), however, a direct comparison between the two sets of data shows excellent agreement (see Supplementary Fig. 3). The discrepancy in the value of *n* simply results from the different *T* ranges of the fits.

**Fig. 5:**
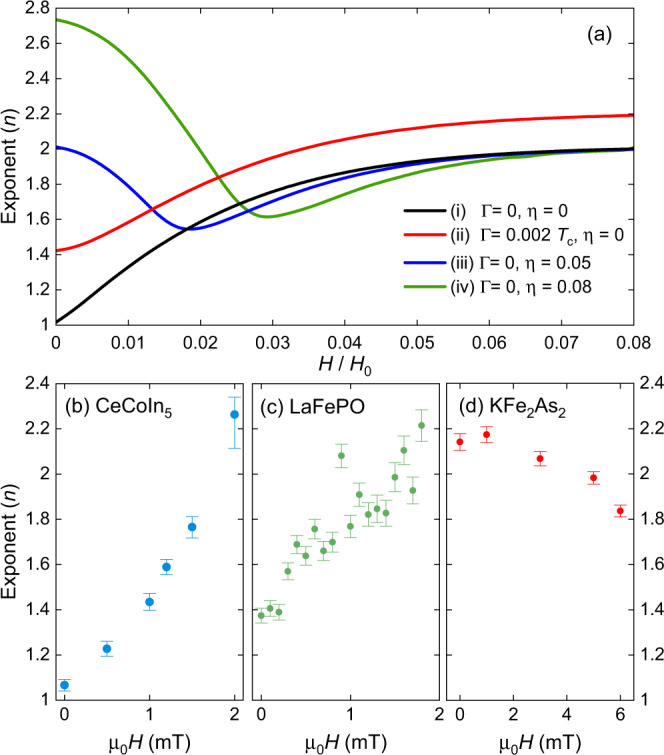
Power law exponent analysis for theory and experimental data. **a** Evolution of the exponent with field from fits to the theoretical response for different gap structures of the form ${{\Delta }}(\phi ,T)={{{\Delta }}}_{0}(T)(| \cos (2\phi )| +\eta )$ and varying impurity concentration Γ (see Supplementary Note 6 for details about the calculations). **b**–**d** Experimental response from CeCoIn_5_, LaFePO and KFe_2_As_2_ respectively. The exponent *n* is extracted from fits to ${\lambda }_{0}^{2}/{[{\lambda }_{0}+{{\Delta }}\lambda (T)]}^{2}=1-A{T}^{n}$ with upper *T* limit of 300 mK for CeCoIn_5_ and KFe_2_As_2_, and up to 400 mK for LaFePO. The values assumed for *λ*_0_ were 190 nm for CeCoIn_5_^40^, 240 nm for LaFePO^13^ and 200 nm for KFe_2_As_2_^41^. For the fits, to the theoretical response in (**a**) an upper limit of *T*/*T*_*c*_ = 0.1 was used. The error bars are the standard error deduced from the fit including a small contribution from the uncertainty in the background determination.

Figure 5b–d shows how the exponent *n* varies with *H* for our three compounds. At zero field, CeCoIn_5_ has *n* close to 1, consistent with line nodes in the clean limit. As *H* increases, *n* increases too and slightly exceeds 2 at the highest field. For LaFePO the behaviour is similar, but with *n* higher than 1 in zero field, consistent with a small number of impurities. Once again, KFe_2_As_2_ shows contrasting behaviour with *n* being close to 2 in zero field and *n* decreasing with increasing *H*. Hence, for CeCoIn_5_ and LaFePO $\tilde{\rho }$ becomes less temperature-dependent at low *T* in the increasing field, whereas for KFe_2_As_2_ the opposite is true.

This experimental behaviour can be compared directly with a similar analysis of our calculations of the non-linear response. In Fig. 5a, we show the evolution of *n* for a nodal gap structure with and without impurities, together with the response for finite-gap structure (with two different values of finite gap). For the pure *d*-wave case (i), *n* increases from 1 to 2 as *H* is increased, with the majority of the increase occurring for *H* ≪ *H*_0_ (*n* ≈ 1.7 for *H*/*H*_0_ = 0.025). The addition of impurities (case (ii)) increases *n*(*H* = 0) and also causes *n* to more slowly approaching its high field limit which is now slightly above 2. Cases (iii) and (iv) simulate two different sizes of finite gaps. In both cases, *n* begins above 2 and then decreases, reaching a minimum before increasing and finally saturating at 2 at high field.

The experimental data for CeCoIn_5_ is close to the clean nodal d-wave response [case (i)] and for LaFePO it is similar to impure nodal response [case (ii)]. For KFe_2_As_2_ the decrease in *n* is again shown to be similar to the finite gap cases (iii) and (iv) although it was not possible to extend the field to high enough values to observe the predicted minimum in *n* because this is limited by *H*_*p*_ or *H*_*c*1_ (see Supplementary Note 4). One drawback to this exponent analysis is that contributions from paramagnetic impurities, or extrinsic paramagnetic particles on the sample or support rod, will add to Δ*λ*(*T*) and be almost field independent for weak-field *H* < *H*_*c*1_ (see Supplementary Note 5). In the nodal case, for *H* = 0 where *dλ*/*dT* is large, a small paramagnetic contribution will increase *n* but may be difficult to distinguish from the case of a small concentration of non-magnetic impurities. However, with increased *H*, *dλ*/*dT* decreases and the relative effect on the paramagnetic impurities will be much larger, leading to an increase in *n* above the limiting value of ~2 in Fig. 5a or even a negative value of *dλ*/*dT* (see Supplementary Note 7).

Our measurements of *λ*(*H*, *T*) in CeCoIn_5_ and LaFePO provide the first unambiguous observation of the theoretically predicted^4,32,33^ field effect on the magnetic penetration depth in nodal superconductors. At the lowest temperatures, Δ*λ*(*H*) is linear-in-*H* and its magnitude decreases strongly with increasing temperature as predicted. Although the effect may be too small to be clearly seen in the cuprates, the response in lower *T*_*c*_ unconventional superconductors is much stronger. This is because the maximum size of *λ*(*H*) is determined by the ratio *H*_*c*1_/*H*_0_ and is approximately proportional to ${T}_{c}^{-1}$ and hence the response is much larger for low-*T*_*c*_ materials than for optimally doped cuprates. Furthermore, the bulk non-linear response in cuprates can be masked by zero-energy surface Andreev bound states^34–36^, which should not be present (at zero energy) in multiband materials with sign-changing gaps such as iron-based superconductors^37^ or CeCoIn_5_.

We have shown how the field dependence of the magnetic penetration depth can be used to distinguish between nodal and small, finite gap structures. The magnetic field causes *λ*(*T*) in the former case to become less temperature-dependent at low temperature (*T* ≪ *T*_*c*_), whereas an increased temperature dependence is found for the latter case. The results confirm that both CeCoIn_5_ and LaFePO have a sign-changing nodal gap structure, whereas for KFe_2_As_2_ the response is dominated by a small but finite gap, restricting possible gap symmetries. With further modelling of the non-linear response for multiband and multigap superconductors in the presence of impurities, it should be possible to extract more quantitative information on the size of the minimum energy gaps and the limits to any possible contribution from sheets with gap nodes. As measurements of the field dependence of *λ* are relatively simple to perform by adding a dc field to the widely used tunnel-diode penetration depth apparatus, we expect that it will prove to be a very useful addition to the toolkit used to investigate gap symmetry in other candidate nodal superconductors.

## Methods

### Sample growth and characterisation

Single crystals of KFe_2_As_2_ were grown by a self flux technique^38^ and are similar to those used in previous heat capacity studies^25,38^. The composition was confirmed by x-ray diffraction and their high quality was evidenced by very narrow specific heat transitions (~0.2 K). We found that the measured *λ*(*T*) was very sensitive to the surface quality and exposing them to air even for a short time changed *λ*(*T*) markedly, producing a fully gapped response. Hence, the samples were cleaved on all six sides in an argon glove box and covered with degassed grease at all times when outside of this. The CeCoIn_5_ single crystal samples were grown in an In flux and composition/structure confirmed by x-ray diffraction. We found that long term exposure to air resulted in slightly visible surface corrosion and so the samples were also cleaved and covered with grease prior to measurement. The LaFePO crystals were grown in a Sn flux and composition/structure confirmed by x-ray diffraction. The samples were not found to be air-sensitive so were not cleaved. Similar crystals were used for prior zero-field *λ*(*T*) and quantum oscillations studies^19,39^.

### Measurements of the magnetic penetration depth

Our measurements of *λ*(*T*, *H*) were performed using a radio frequency (RF) tunnel diode oscillator technique^5^. The sample is attached to a sapphire cold finger and inserted into a small, copper solenoid which forms part of a tank circuit that is resonated at 14 MHz using the tunnel diode circuit. The sapphire cold finger is cooled down to a minimum temperature of 60 mK using a dilution refrigerator. The RF field is estimated to be 0.2 μT and the Earth’s field is shielded using a mu-metal can.

For the field-dependent measurements, a small dc field is applied parallel to the RF field using a superconducting solenoid. The samples are first cooled to base temperature (~60 mK) in zero field before applying the dc field in order to minimise any effect of vortices entering the sample and contributing to *λ*(*T*). The samples were then warmed to 0.3 K (0.6 K for LaFePO) and cooled back to a base temperature several times to average the data and remove any thermal drift. The effects of dc field on the weak background paramagnetism from the sapphire rod are close to the noise level and are neglected (see Supplementary Note 1).

In each case, we set the zero for Δ*λ*(*T*) to be the lowest temperature (${T}_{\min } \sim 50$ mK) for the zero-field measurement, i.e. ${{\Delta }}\lambda ({T}_{\min },H=0)=0$. The measurements in finite field are then shifted in *λ* so that they coincide with the zero-field measurements at *T* = 0.3 K (or *T* = 0.6 K for LaFePO). This process is necessary because applying a dc field causes a shift in the frequency of the resonant circuit which is not due to the sample and is difficult to subtract reliably. By fixing the dc field and sweeping the sample temperature only, the dc field response of the coil is not measured.

## Supplementary information

Supplementary Information

## Data Availability

The datasets generated during and/or analysed during the current study are available from the corresponding author on reasonable request.
