# Supplementary material for: Observation of the non-linear Meissner effect
Source: Nat Commun. 2022 Mar 7;13:1201. doi: 10.1038/s41467-022-28790-y (PMC8901765; doi:10.1038/s41467-022-28790-y)
Supplement: Supplementary file 1 — Supplementary Information [file 41467_2022_28790_MOESM1_ESM.pdf]

Observation of the Non-linear Meissner Effect: Supplementary Information

J. A. Wilcox,[1] M. J. Grant,[1] L. Malone,[1] C. Putzke,[1,*] D. Kaczorowski,[2] T. Wolf,[3] F. Hardy,[3] C. Meingast,[3] J. G. Analytis,[4,5,†] J.-H. Chu,[4,5,‡] I.R. Fisher,[4,5] and A. Carrington[1,§]

[1]*H. H. Wills Physics Laboratory, University of Bristol, Tyndall Avenue, Bristol, BS8 1TL, United Kingdom*
[2]*Institute of Low Temperature and Structure Research, Polish Academy of Sciences, 50-950 Wroclaw, Poland*
[3]*Institute for Quantum Materials and Technologies, Karlsruhe Institute of Technology, 76021 Karlsruhe, Germany*
[4]*Geballe Laboratory for Advanced Materials and Department of Applied Physics, Stanford University, California 94305-4045, USA*
[5]*Stanford Institute for Materials and Energy Sciences, SLAC National Accelerator Laboratory, 2575 Sand Hill Road, Menlo Park, California 94025, USA*

Supplementary Note 1. Setup and Background

In our measurements the sample is mounted, with silicone grease, on a sapphire rod which protrudes into the RF coil. The dc field coil is co-linear as shown along with the rest of the setup in Fig. 1. The sample on the sapphire rod may be moved within or withdrawn completely from the coils at low temperature to facilitate calibration, measurement of field dependent background or to vary the RF field on the sample. The size of the RF field was measured to be $\sim 0.2\mu$T. The thermometer is mounted on the other end of the sapphire rod. We have estimated the RF heating P_{RF} of our sample of $CeCoIn_5$ at $T \ll T_c$ using data for the surface resistance in Ref. [1] extrapolated to our measurement frequency. We find that $P_{RF} \simeq 10^{-17}$ W and combining this with the thermal resistance of the sapphire rod and the interface thermal

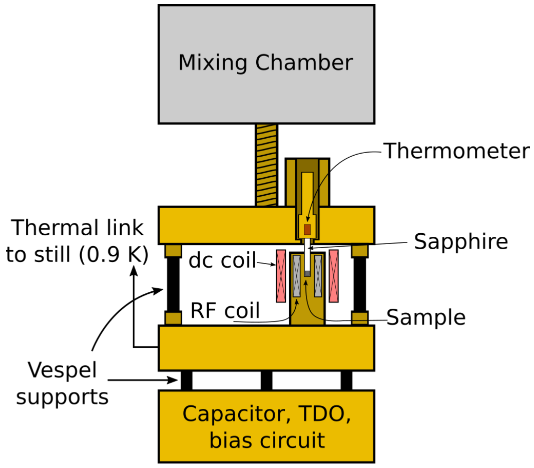

Supplementary Fig. 1. Schematic of experimental setup.

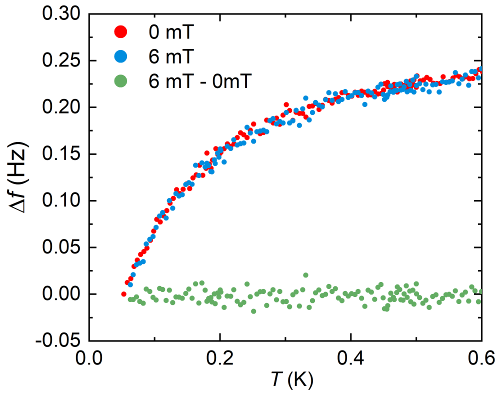

Supplementary Fig. 2. Effect of *dc* field on the background from the sample support rod. Data are shown for dc fields $H = 0$ and $H = 6$ mT, together with the difference between the two data sets.

resistance between the sample and rod [2], we estimate that at ~ 100 mK the RF heating raises the temperature of the sample by less than 1 nK and is therefore negligible.

The temperature dependent background due to the very weak paramagnetism of the sapphire rod is measured separately with the sample removed from the platform. This background fits a Curie-Weiss form. The effect of dc field on this background (Fig. 2) is close to zero within the noise level.

Supplementary Note 2. Sample Dimension

All three compounds, $CeCoIn_5$, LaFePO and KFe_2As_2 have a tetragonal structure and the single crystal samples in this study took the form of thin platelets, with the shortest dimension (l_z) corresponding to the c axis and the larger dimensions (l_x and l_y) corresponding to the a-b plane. The dimensions of all samples are listed in Table 1.

Supplementary Note 3. Measurement geometry

For thin, platelet samples measurements of the in-plane penetration depth can be conducted in two geometries. With the field directed along the short c-axis the screening currents

---

* Present address:Laboratory of Quantum Materials, Institute of Materials, École Polytechnique Fédérale de Lausanne (EPFL), 1015 Lausanne, Switzerland
† Present address:Department of Physics, University of California, Berkeley, California 94720, USA.
‡ Present address: Department of Physics, University of Washington, Seattle, WA, 98195 USA
§ Corresponding author: email a.carrington@bristol.ac.uk

Sample	l_x (μm)	l_y (μm)	l_z (μm)
$CeCoIn_5$	200	310	5
LaFePO	270	274	31
KFe_2As_2	715	405	50

Supplementary Table 1. The dimensions of the samples used in this study.

flow only in the ab-plane, but the demagnetising effects will be large, with the field strongly enhanced on the ac or bc faces of the crystal. The field enhancement is particularly large close to the corners, and so the field will be quite non-uniform (see below for details of this). This is clearly not ideal for our measurements where we are concerned with the field dependence of λ. The alternative geometry is where the field is directed along the ab-plane. In this case, the demagnetisation effects are very small, and the field uniform, but the measured $\lambda_m(T)$ will be a mixture of the in-plane and the c-axis response. To a good approximation, the volume penetration by field in the $H\|a$ geometry will be given by

$$\Delta V = 2(\ell_a \ell_b \lambda_{ab} + \ell_a \ell_c \lambda_c),$$

where ℓ_a, ℓ_b, ℓ_c are the physical dimensions along each axis. If we then calculate an effective λ from ΔV by dividing by the in-plane dimensions we have

$$\lambda_e = \lambda_{ab} + \frac{\ell_c}{\ell_b}\lambda_c.$$

As long as the samples are sufficiently thin ($\ell_c/\ell_b \ll 1$) or more precisely $(\ell_c \Delta\lambda_c(T))/(\ell_b \Delta\lambda_{ab}(T)) \ll 1$ then λ_e is very close to λ_{ab}. Demagnetising effects are avoided and the measured $\Delta\lambda(T)$ is predominately the in-plane response.

For $CeCoIn_5$ and KFe_2As_2, the large aspect ratios led us to use the $H\|ab$ geometry for our measurements in order to minimise demagnetising effects, as well as any contributions from non-local effects or Andreev bound states. For LaFePO the thicker samples and larger λ_c/λ_a anisotropy meant the the $H\|c$ geometry was more suitable.

For $CeCoIn_5$, Howald *et. al.*[3] report an anisotropy in the zero temperature penetration depth $\gamma = \lambda_c/\lambda_a \simeq 1.3$ and over the temperature range $T = 0$ to $T/T_c = 0.25$, the ratio $\Delta\lambda_c/\Delta\lambda_a \simeq 4.4$. Our sample has an aspect ratio of approximately 50 (Table S1), which means that the c-axis contribution to λ_e would be $\sim 10\%$. However, Howald *et. al.* find a much weaker temperature dependence of $\Delta\lambda_a$ compare to our current data, possibly because of sample purity issues. If we use our own data for $\Delta\lambda_{ab}(T)$ in conjunction with $\Delta\lambda_c(T)$ from Howald *et. al.* the c-axis contribution drops to around 2%. In both cases this is a relatively small correction.

In the case of KFe_2As_2, we are not aware of direct measurements of $\lambda_c(T)$. However, the anisotropy in the absolute values at $T = 0$ has been measured to be relatively weak, with $\gamma = 3.6$ [5]. In Supplementary Fig. 3, the response for our KFe_2As_2 sample, in the $H\|ab$ orientation, is shown in direct comparison to the in-plane response $\Delta\lambda_{ab}(T)$ from Ref. [4]. The agreement between the two measurements is very good and shows that there is very little c-axis contribution to the response over the temperature range in question.

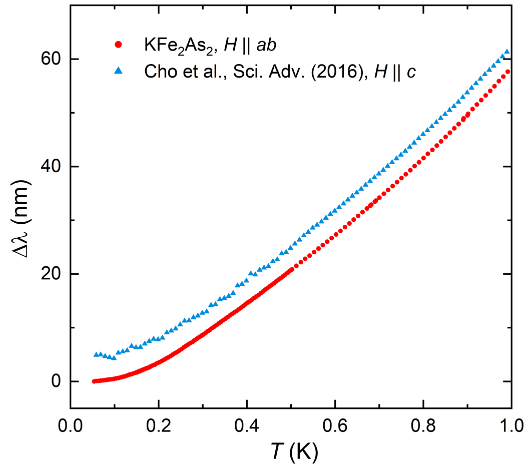

Supplementary Fig. 3. Comparison between measurements of $\Delta\lambda(T)$ for KFe_2As_2. Our data for KFe2As2, measured in the $H\|ab$ orientation is compared to the in-plane response reported in Ref. [4] (offset by 5nm along the $\Delta\lambda$ axis for clarity).

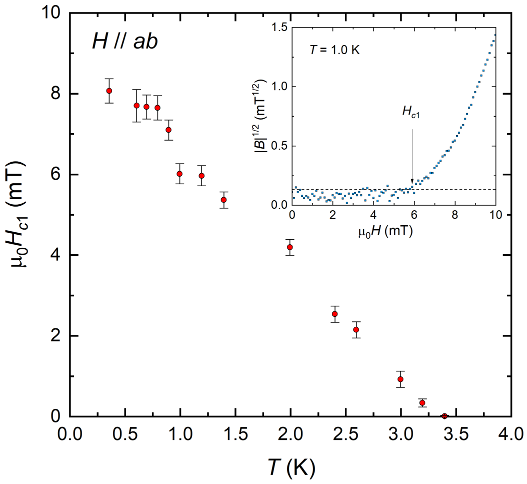

Supplementary Fig. 4. Temperature dependence of the lower critical field H_{c1} of KFe_2As_2. For these measurements the magnetic field is oriented in-plane ($H\|ab$). The inset shows an example response of a Hall sensor located beneath the edge of the sample. H_{c1} is the field at which the measured flux density begins to increase. The error bars in the main figure reflect the uncertainty in determining H_{c1} from plots similar to the inset.

Supplementary Note 4. Determination of Field of First Flux Penetration

For KFe_2As_2 and LaFePO, the lower critical field H_{c1}, or more precisely the field of first flux penetration H_p, was determined in the same samples used for the $\lambda(T, H)$ study. The samples were suspended above a micro-Hall probe array, cooled to base temperature and then a dc field was applied as described in Ref. [6]. For KFe_2As_2, H was parallel to the ab-plane and for LaFePO $H\|c$ as in the $\lambda(T, H)$ study. At low field the local field measured by the Hall probe is linear in H corresponding to the field leaking around the sides of the

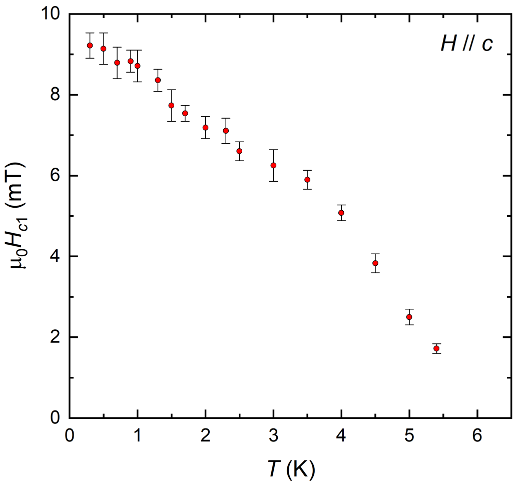

Supplementary Fig. 5. Temperature dependence of the lower critical field H_{c1} of LaFePO. In these measurements the magnetic field is oriented parallel to the c-axis ($H \| c$). Note the field values here have been corrected for demagnetising effects which increases the effective field by a factor ∼5, so the actual applied field which would exceed H_p is ∼2 mT. The error bars include the uncertainty in determining H_p (as in Supplementary Fig. 4) combined with the uncertainty in the demagnetising factor from the uncertainty in the sample dimensions.

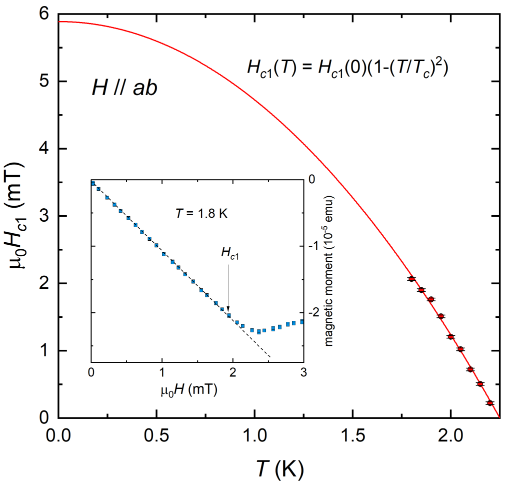

Supplementary Fig. 6. Temperature dependence of the lower critical field H_{c1} of CeCoIn$_5$. For these measurements the magnetic field is oriented in-plane ($H \| ab$) and the values of H_{c1} are determined from bulk magnetisation using a SQUID magnetometer. The inset shows an example measurement for positive field, where H_{c1} is taken as the field at which the susceptibility χ deviates from -1 (as indicated by the dashed line). The error bar in the main figure reflect the uncertainty in determining H_{c1} from plot similar to the inset.

sample. At H_p there is a sharp rise in B as seen in the inset to Supplementary Fig. 4. This field, which is equal to H_{c1} in the absence of any surface barriers is plotted in Figs. 4 and 5. $H_{c1}(T)$ is found to have a linear temperature dependence, mirroring that found for $1/\lambda(T)^2$. For LaFePO the field has been corrected for demagnetising factors using the method of Brandt [6, 7]. As the fields are small, sweeps were made in positive and negative fields to correct for the Earth's field.

For CeCoIn$_5$, a different method was used because of the unavailability of apparatus. Instead, H_{c1} was measured using a commercial SQUID magnetometer. The magnetic moment was measured as a function of field and the point where $m(H)$ departs from linearity identified as H_{c1} (see insert to Supplementary Fig. 6). The data only extend down to $T = 1.7$ K and so were extrapolated to lower temperature using the standard empirical formula $H_{c1}(T) = H_{c1}(0)(1 - (T/T_c)^2)$. Although this extrapolation is only appropriate for conventional fully gapped superconductors, a linear extrapolation as suggested by the linear T dependence of $1/\lambda^2(T)$ would give a higher value of $H_{c1}(0)$, so the extrapolation in Supplementary Fig. 6 ($H_{c1}(0) = 5.9 \pm 0.1$ mT) should be viewed as a lower limit. The results presented are similar to those found in the literature; performing the same analysis on the values of $H_{c1}(T)$ reported by Majumdar *et. al.*[8] yields a result of $H_{c1}(0) = 4.52 \pm 0.02$ mT.

Supplementary Note 5. Other contributions to $\lambda(T, H)$: Paramagnetic impurities, and Andreev bound states

Impurities, both magnetic and non-magnetic can affect the measured behaviour of $\lambda(T, H)$ as can surface Andreev bound states. In this section we consider the effect of paramagnetic impurities and Andreev bound-states. Non-magnetic impurities are considered in the next section.

Paramagnetic impurities can contribute to the measured penetration depth, giving a contribution which is proportional to the normal state susceptibility $\chi_N(T)$ [9, 10]. A flattening or an upturn at low temperatures in $\lambda(T)$ can then result from $\chi_N(T)$ following a Curie-law behaviour if the concentration of paramagnetic ions is sufficiently large. The effect of finite field on these paramagnetic terms can be estimated from the calculated field dependence of $\chi_N(T)$.

For classical paramagnetism, the induced magnetisation M in a magnetic field B is given by

$$M = N g_J \mu_{\rm B} J F_J(x), \tag{1}$$

where N is the number of moments per unit volume, g_J is the Landé g-factor, J is the total electronic angular momentum and $F_J(x)$ is the Brillouin function,

$$F_J(x) = \frac{2J+1}{2J} \coth\left(\frac{2J+1}{2J}x\right) - \frac{1}{2J}\coth\left(\frac{1}{2J}x\right), \tag{2}$$

in which x is defined as

$$x = \frac{J g_J \mu_{\rm B} B}{k_{\rm B} T}. \tag{3}$$

From this we can calculate the susceptibility

$$\chi = \frac{dM}{dH} = \frac{N\mu_0(g_J\mu_B J)^2}{k_B T}\frac{dF_J(x)}{dB} \tag{4}$$

The susceptibility of these moments will then contribute to the measured penetration depth $\lambda_m(T)$ according to

$$\lambda_m(T) \simeq \lambda_L(T)\sqrt{1+\chi(T)}, \tag{5}$$

where $\lambda_L(T)$ is the London penetration depth (i.e. no impurities) [9]. For small amounts of impurities, $\chi \ll 1$, the change in the penetration depth with temperature can be expressed as [10]

$$\Delta\lambda(T) = \Delta\lambda_L(T) + \chi(T)\lambda_0/2. \tag{6}$$

As an example, we consider the case of Ce^{3+} ($S = 1/2, J = 5/2$), which is a plausible impurity in $CeCoIn_5$. We assume a pure linear dependence of $\Delta\lambda_L(T)$ and add a small paramagnetic contribution which, in the limit of zero applied field, gives a characteristic flattening of $\Delta\lambda(T)$ at low-T (Supplementary Fig. 7). Increasing the field to 3 mT (maximum experimental value for $CeCoIn_5$), gives a result almost indistinguishable from the zero field curve. A further increase to $\mu_0 H = 30$ mT gives a very small decrease in $\Delta\lambda$ of approximately 0.2 nm at 50 mK. Only with a field 100 times larger than used experimentally (300 mT) is a significant response induced, and in that case the effect is to decrease $\Delta\lambda$, i.e., the change is in the opposite direction to that observed for $CeCoIn_5$. This large difference in field scale between the effect of finite field on paramagnetic impurities compared to that for the non-linear-Meissner effect is the key point which allow us to distinguish between the two effects. Note that for lower spin species (e.g., $J = 1/2$) the effect would be even weaker.

Andreev bound states (ABS) can give an additional contribution to the low temperature penetration depth. In the case of the cuprates, where there is a single sign-changing gap due to the $d_{x^2-y^2}$ pairing state, zero energy ABS occur on sample surfaces which are not perpendicular to the principal crystallographic directions; (100), (010), or (001) [11, 12]. These zero energy states are broadened in energy at finite temperature and contribute a zero energy peak to the density of states and hence give rise to an upturn in $\lambda(T)$ at low temperatures if the field is probing ABS surfaces [13–15]. This upturn is suppressed in small fields because the ABS split and shift higher in energy with increasing field [13, 15]. This increase in temperature dependence of $\lambda(T)$ with increasing field is opposite to what we find for $CeCoIn_5$ or LaFePO and hence any ABS contribution must be small compared to the intrinsic bulk non-linear response. This is consistent with the $H\|ab$ measurement geometry used for $CeCoIn_5$. With this geometry the main contribution to $\Delta\lambda(T)$ comes from the (001) faces on which ABS cannot form. ABS contribution were not detected for $YBa_2Cu_3O_{6+x}$ in this geometry for the same reason [15].

For iron-based superconductors with a sign-changing gap, theory suggests that any ABS would occur at finite energy [16, 17] and so would not produce a zero-energy peak in

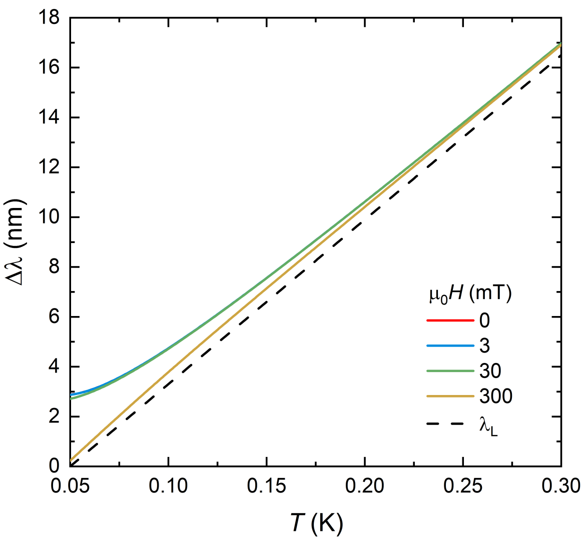

Supplementary Fig. 7. Calculation of the expected change in the magnetic penetration depth due to paramagnetic impurities. The response of Ce^{3+} impurities in fields of 0 (red curve), 3 mT (blue curve), 30 mT (green curve) and 300 mT (yellow curve), compared to a pure linear response (dashed black line).

the DOS and hence would not give rise to an low-T upturn in $\lambda(T)$ or the associated field dependence. For LaFePO, the measurement geometry $H\|c$ would in-principle allow for ABS contributions as the sample has both (100) and (110) facets [18]. However, the observed increase in $d\lambda/dT$ with increasing field would suggest that any contribution was either small or entirely absent. For KFe_2As_2 the measurement geometry, $H\|ab$, means predominately (001) surfaces are probed and this together with the above mentioned theoretical work suggests that there are also no ABS contributions.

Supplementary Note 6. Calculations of $\lambda(T, H)$

For our calculations of the field dependence of $\lambda(T)$ we consider a two-dimensional, circular Fermi surface with isotropic Fermi velocity v_F. The gap structure is taken as either the classic d-wave form

$$\Delta(\phi, T) = \Delta_0(T)\cos(2\phi) \tag{7}$$

or a similar form but with a finite gap

$$\Delta(\phi, T) = \Delta_0(T)(|\cos(2\phi)| + \eta). \tag{8}$$

Note that $\lambda(T, H)$ is only sensitive to the absolute magnitude of $\Delta(\phi)$, not its sign. The second, finite gap form could represent a mixed order parameter $d + is$ but is also representative of other strongly anisotropic forms where Δ does not change sign. ϕ is the azimuthal angle. The temperature dependence of the gap is taken to be the weak-coupling d-wave form in both cases, which is approximated by

$$\Delta_0(t) = \Delta_0(0)\tanh\left[\frac{\pi}{\Delta_0(0)}\sqrt{\frac{4}{3}\left(\frac{1}{t}-1\right)}\right] \tag{9}$$

where $\Delta_0(0) = 2.14$ and $t = T/T_c$.

To calculate $\lambda(t, H)$ in the local limit, we used the following expression [19, 20] for the non-linear quasiparticle current j_{qp} in the x-direction ($\phi = 0$) created by the applied field, integrated over the Fermi surface

$$j_{qp} = A \int_{-\pi}^{\pi} d\phi \mathcal{R} \sum_n \frac{(\sigma(\phi) - i\omega_n)\cos(\phi)}{\sqrt{(\omega_n + i\sigma(\phi,t))^2 + |\Delta(\phi)|^2}} \quad (10)$$

where $\omega_n = (2n+1)\pi t$ are the Matsubara frequencies,

$$\sigma(\phi) = \alpha H \cos(\phi - \phi_0) \quad (11)$$

is the quasiparticle energy shift produced by the field H induced superflow and $\mathcal{R}$ denotes the real part of the sum. The limits of the sum over n are $\pm\infty$ but practically we set a maximum frequency $|\omega_0| = 100$ (in units of T_c). Convergence was checked by varying $|\omega_0|$ up to 2 orders of magnitude higher and checking the H and T dependent results were insensitive. The direction of the field is set by ϕ_0. A is a constant related to the normal state parameters which determine the absolute value of $\lambda(0)$, and $\alpha = \mu_0 v_F \lambda(0) e/(k_B T_c)$ is the constant of proportionality between the field and energy shift (normalised to $k_B T_c$).

The temperature and field dependence of the normalised superfluid density is then calculated from

$$\frac{\lambda^2(0)}{\lambda^2(t,H)} = C\frac{j_{qp}}{H}, \quad (12)$$

where C is a constant determined by ensuring $\lambda^2(0)/\lambda^2(t,H) = 1$ as $(t,H) \to 0$. This represents an approximation which is exact in the linear-response limit, and gives the correct slope $\mathrm{d}\lambda/\mathrm{d}t = \ln 2/(\Delta_0/T_c)$ as $(t,H) \to 0$ [20] for the d-wave gap (Eq. 7). To get the exact result for finite H, the non-linear London equations should be solved, combining the result for q_{qp} with Maxwell's equations, in the appropriate geometry. However, it was shown in Refs. [19, 20] that in the usual semi-infinite plane geometry this only changes the field scale by a factor 3/2 without otherwise changing the field or temperature dependence of λ. Our approach mirrors that of Xu *et al.* [20] who solved the non-linear London equations analytically for the pure, zero temperature field dependence of λ, but for finite temperature/scattering they calculated numerically the current-field relation and scaled this to the pure, $T = 0$ result to get the non-linear response of λ. Our finite H results for $\lambda(H)$ are consistent with those of Stojković and Valls [19] who did solve the non-linear London equations at finite T.

The results for the two different gap forms, d-wave gap (Supplementary Eq. 7) and $(d + is)$-gap [Supplementary Eq. 8 with $\eta = 0.2$] in the pure limit ($\Gamma = 0$) are shown in Supplementary Fig. 8 (a) and (b). The magnetic fields in the calculations are scaled according to

$$\mu_0 H_0 = \frac{3\Delta_0(0)}{e\lambda(0)v_F} \quad (13)$$

which takes into account the above factor 3/2. In the main text we subtract a constant from each curve at finite H so that $\Delta\lambda(t)$ coincides with the $H = 0$ result at $t = 0.2$ for easy comparison with the experimental data.

Non-magnetic impurities are included by replacing the Matsubara frequencies ω_n with their renormalised values

$$\tilde{\omega}_n = \omega_n + \Gamma \frac{N(\tilde{\omega}_n, t)}{c^2 + N^2(\tilde{\omega}_n, t)} \quad (14)$$

where

$$N(\omega, t) = \frac{1}{\pi}\int_{-\pi/2}^{\pi/2} \frac{\omega d\phi}{\sqrt{\omega^2 + \Delta^2(\phi, t)}} \quad (15)$$

is the quasiparticle density of states, c is the cotangent of the scattering phase shift and $\Gamma = (1 + c^2)\Gamma_N$, where Γ_N is the normal state scattering rate [20]. Equations (14) and (15) are solved iteratively until convergence is reached for each Matsubara frequency, *i.e.*, $N(\omega_n, t)$ is first calculated with the unrenormalised ω_n, then $\tilde{\omega}_n$ is calculated from Supplementary Eq. (14) and this is used to calculate $N(\tilde{\omega}_n, t)$. This is repeated until $\tilde{\omega}_n$ has converged. The reduction of the superfluid density with increasing Γ_N calculated by this procedure as $(t, H) \to 0$ is in excellent agreement with the results of Deepwell *et al.* [21] for both the unitary and Born limits. Our calculations of $\lambda(T, H, \Gamma)$ are in good agreement with the results of Refs. [20] for the unitary limit ($c = 0$) case considered there.

Results are shown in Supplementary Fig. 9 for the d-wave gap function and two different values of Γ to illustrate small and large amounts of impurity scattering in the unitary limit respectively. It can be seen that for a small impurity density, similar to that which would cause a flattening of $\Delta\lambda(T)$ at very low temperature only, the effect on the non-linear response is quite small. The normalised $\Delta\lambda(T)$ still increases with increasing field. Only when the impurity scattering is large, so that $\lambda(T) \sim T^2$ over the whole low temperature range is the non-linear response substantially reduced.

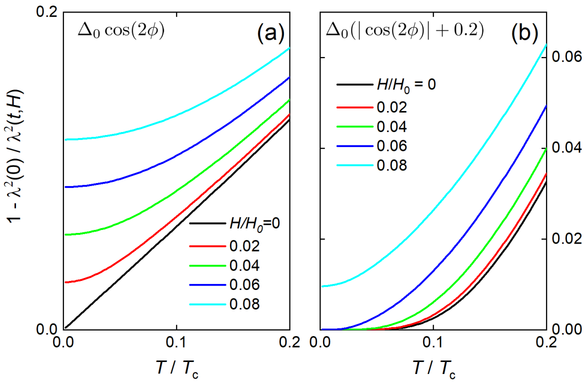

Supplementary Fig. 8. Calculated temperature dependence of the normalized superfluid density $\lambda^2(0)/\lambda^2(t, H)$. The fields used in the calculations are indicated (in units of H_0) and the calculations are in the pure limit (no impurities). (a) d-wave gap (Eq. 7) (b) $(d + is)$-gap (Eq. 8 with $\eta = 0.2$). This is the same as figure 4 of the main text, but without the results being shifted to coincide at $T/T_c = 0.2$.

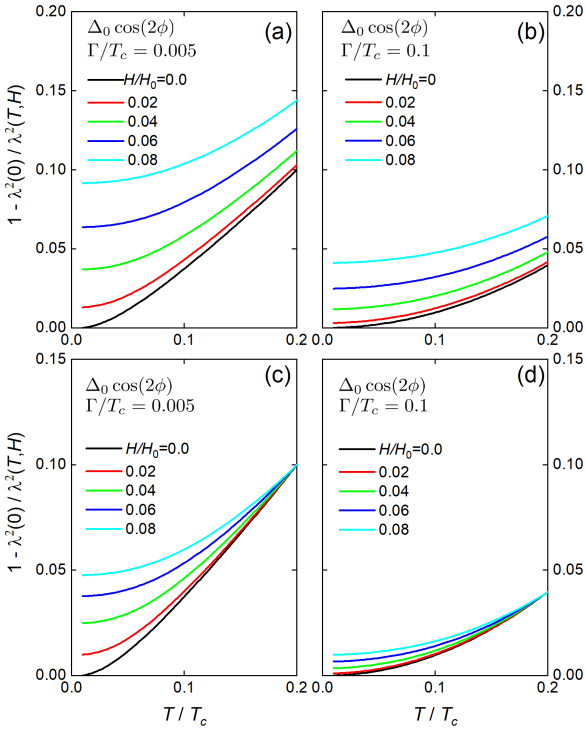

Supplementary Fig. 9. Effect of impurities on the non-linear response for a d-wave gap. (a) low impurity density, $\Gamma = 0.005$, (b) high impurity density, $\Gamma = 0.1$. (c) and (d) are the same data shifted vertically down so that they coincide with the $H = 0$ results at $T/T_c = 0.2$ as in figure 4 of the main text.

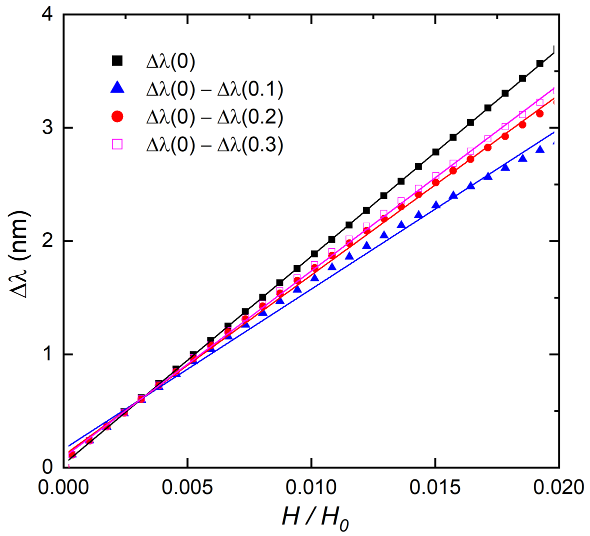

Supplementary Fig. 10. Calculated field dependence at fixed temperature. Calculated change in λ with field at $t = T/T_c = 0$ (black squares), for a $d-$wave gap, showing the expected linear response. Also shown is this zero temperature result with $\Delta\lambda(H)$ at higher temperatures ($t = 0.1, 0.2, 0.3$) subtracted. The solid lines are linear fits to each data set. $\lambda(T = 0, H = 0)$ has been set to 240 nm which is the value appropriate for LaFePO.

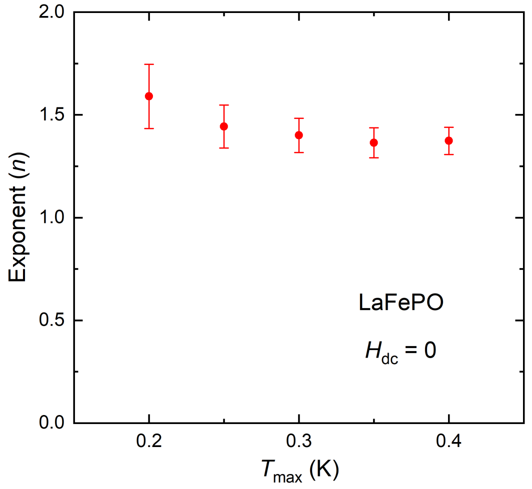

Supplementary Fig. 11. Effect of varying the upper temperature limit of the fit on the exponent n found for LaFePO. The minimum temperature was kept fixed at the base temperature. The error bars are the standard error from the fit.

Field dependence of λ at fixed temperature. In our analysis in the main text, (Figure 2) we have shifted the field dependent $\lambda(T, H)$ data so that the results for each T sweep coincide at $T = 0.3$ K ($CeCoIn_5$ and KFe_2As_2) or $T = 0.6$ K (LaFePO) in order to remove the field dependent (but temperature independent) background from the measurement coil, and also to emphasise the change in temperature dependence. This is also done for the comparison theory curves (main text, figure 4). In Supplementary Fig. 10 we show the the calculated field response of a clean, d-wave superconductor at $T = 0$, with the response $\Delta\lambda(H)$ at higher temperatures subtracted. This simulates the analysis shown in figure 3 of the main paper, with parameters (maximum field and λ_0) taken to correspond approximately to our results for LaFePO. As temperature is increased the theory predicts that the low field response is substantially reduced, so that for weak fields, subtracting the high temperature response from the zero temperature one has only a small effect on $\Delta\lambda(H)$. Although, this subtracted $\Delta\lambda(H)$ is no longer strictly linear in H it can be seen that the non-linearity is weak, with the main effect being a small decrease in slope.

Supplementary Note 7. Exponent analysis

Whether the shifted $\Delta\lambda(T, H)$ (as in main text Fig. 2) increases or decreases with H at the lowest temperatures for the case of a finite gap ($\eta > 0$) depends on the size of the field with respect to the size of the finite gap and also the temperature at which the data are shifted to coincide. An alternative way to view the results is to fit the data to a power-law as in Supplementary Fig. 5 of the main text. As the data do not precisely follow a power-law over the full temperature range of the fit, the derived exponent will be a temperature-averaged value which depends slightly on the upper temperature limit of the fit. An example of the dependence of n on the upper T

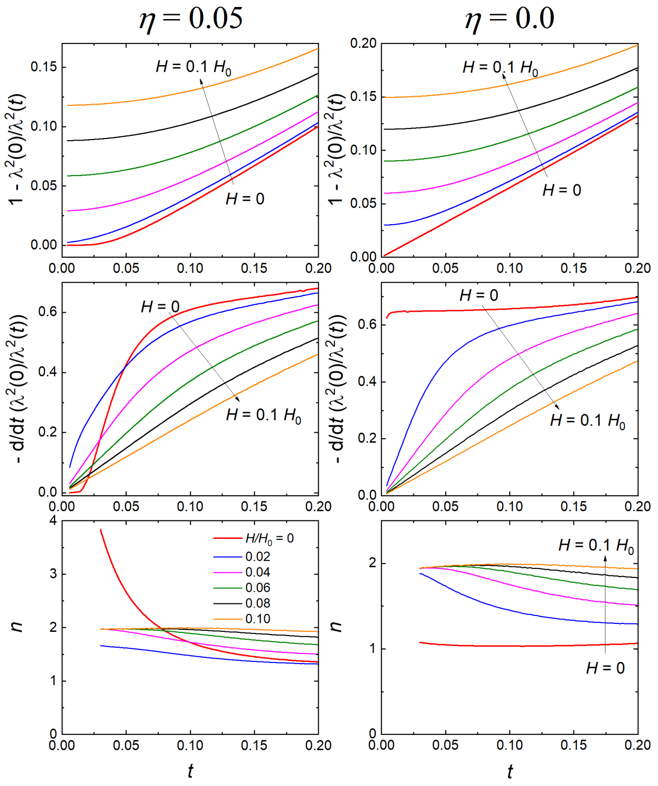

Supplementary Fig. 12. Exponent analysis of the calculated superfluid density. Left panels for the small gap case ($\eta = 0.05$) and right panels for the pure-d-wave case ($\eta = 0$). Top panels: $\lambda^2(0)/\lambda^2(t, H)$ for the fields indicated. Middle panels: the derivative $\mathrm{d}\lambda^2(0)/\lambda^2(\mathrm{t},\mathrm{H})/\mathrm{dt}$. Bottom panels: The local exponent $n = \mathrm{d}\ln(\lambda(\mathrm{T}) - \lambda(0))/\mathrm{d}\ln\mathrm{T}$.

limit, with the lower T limit always fixed to the base temperature 65 mk is shown in Supplementary Fig. 11). The exponent increases slightly as T_{min} is lowered as expected from $\lambda(T)$ crossing over from $\sim T$ to $\sim T^2$ at lower temperature.

A second alternative is to calculate the local temperature exponent, from $n = \mathrm{d}\ln(\lambda(\mathrm{T}) - \lambda(0))/\mathrm{d}\ln\mathrm{T}$. The result of doing this for the calculation with a very small gap, $\eta = 0.05$, is shown in Supplementary Fig. 12. For zero field, n at low temperature tends to a high value indicative of an exponential T dependence of λ as expected from the model. For $H/H_0 = 0.02$, n has decreased to ~ 1.5 at $t = 0.03$, consistent with a much stronger (almost linear) T dependence of λ. For larger fields, n tends to 2 at the lowest temperatures which is the same as expected for the nodal case with impurities. The derivative $\mathrm{d}\lambda^{-2}/\mathrm{d}T$ shows consistent behaviour, increasing from zero for zero field (exponential behavior) to a maximum for $H/H_0 \simeq 0.02$ and then reduces monotonically for higher fields. For this gapped case, the temperature dependence at the lowest temperature is always enhanced by field at the lowest temperatures with the maximum enhancement occurring for a particular range of small applied field. For the nodal case ($\eta = 0$) (right panels Supplementary Fig. 12), the low temperature exponent is close to 1 at zero field, and then increases monotonically to 2 at higher fields. Unfortunately,

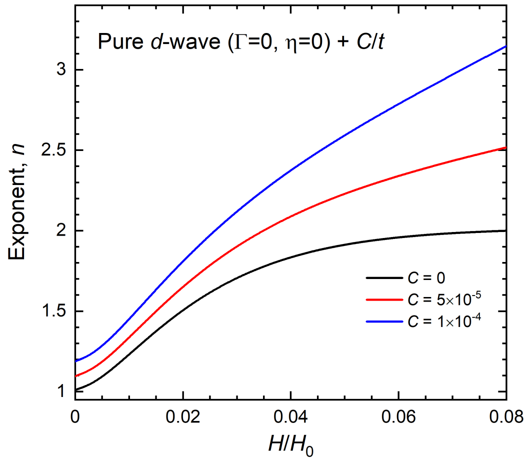

Supplementary Fig. 13. Effect of paramagnetic impurities on the exponent analysis of the calculated superfluid density. The curves show the evolution of the fitted exponent n versus field for the pure d-wave case and with a small field-independent paramagnetic contribution C added. Two different values of C are shown to illustrate the progression of the effect. For all three cases, the calculated superfluid density is fitted between t=0.015 and 0.1, similar to the range used for the fits to the experimental data (main text Fig. 5).

the noise level is too great to perform a similar analysis on the experimental data, so we use the power-law fitting over an extended temperature range method in the main text.

As described in Section (E) above, if the normal state of the superconductor is paramagnetic there will be an additional contribution to $\Delta\lambda(T)$. This will reduce $d\lambda(T)/dT$, even leading to it being negative at sufficiently low temperatures. The effect will be seen most clearly when the intrinsic $d\lambda(T)/dT$ is low, which is the case when there is a finite gap, impurities or at large H. As this contribution will be largely independent of field, in the case of a nodal superconductor, at low fields it will manifest as small increase in n, but at larger fields n can be increased markedly or even make $d\lambda(T)/dT$ become negative. We have illustrated this effect by adding in a small paramagnetic contribution to the theoretical results for the pure d-wave response (Supplementary Fig. 8). The changes in the exponent n versus field for zero and two different sized paramagnetic contributions are shown in Supplementary Fig. 13. The impurities produce only a small change in n at $H = 0$ but at higher fields, where for no impurities $n \to 2$, the effect of impurities on n is much larger as expected.

Supplementary Note 8. Non-local effects

In nodal superconductor, it has been predicted that for Cooper pairs with momentum close to a node the coherence length $\xi \sim v_F/\Delta$ diverges as Δ tends to zero causing a crossover to non-local electrodynamics [22]. In zero field this is predicted to cause $\lambda(T)$ to cross over from T to T^2 below a certain temperature $T^{\star}$, an effect which is similar to that of impurities. The effect of this on the non-linear response has

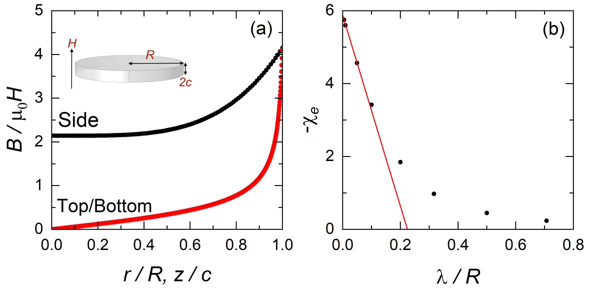

Supplementary Fig. 14. Effect of demagnetisation on response. (a) The calculated variation of the tangential surface fields along the sides and top/bottom faces. The inset shows the geometry of the disc sample for the simulation. (b) The calculated change in the effective susceptibility χ_e as a function of λ.

been calculated by Li *et al.* [23] who find that the non-locality causes $\lambda(H)$ to increase less rapidly than linear below a certain cross-over field, in a similar way to impurities.

In a quasi-two-dimensional superconductor, whether the non-local effect is manifest or not is a strong function of the measurement geometry. In 2D ($\xi_c = 0$), the Cooper pairs are confined to the same plane so if the decrease in B from the Meissner effect is perpendicular to this plane, which is the case when $H \| ab$, there will be no non-local effects. The non-local effects would then only be manifest in the $H \| c$ geometry where B varies over the size of the Cooper pair. As far as we know, this field direction dependence of the *in-plane* penetration depth λ_{ab} has never been seen, although Chia *et al.* [24] suggested the different T dependencies of the in-plane and out-of-plane $\lambda(T)$ in their samples of $CeCoIn_5$ might be caused by this effect. In our experiments, $CeCoIn_5$ was measured in the $H \| ab$ geometry which should minimise any non-local contributions and indeed we found $\lambda(T)$ was closer to T rather than T^2. For these reasons we would not expect non-local effects for our $CeCoIn_5$ measurements. For LaFePO, non-local effects are possible because of the $H \| c$ measurement geometry, although the linear H dependence of $\Delta\lambda(H)$ suggests they are small.

Supplementary Note 9. Demagnetisation effects

As discussed above, for a platelet sample in the $H \| c$ geometry, demagnetisation effects cause the surface fields to be non-uniform. To estimate the effect of this on the measured field dependence of λ we have performed finite-element modelling to determine the surface fields. We chose a simplified geometry of a disk with large aspect ratio ($R : c$ = 10:1) similar to that of our LaFePO sample (see Table 1 and Supplementary Fig. 14(a)). We use the COMSOL package to solve the *linear* London equation inside the disc and Ampere's outside. We solve for the vector potential $\boldsymbol{A}$ subject to the boundary condition that at large distances the B field is uniform and directed along the z-direction.

The variation of the fields along the side and top/bottom faces are shown in Supplementary Fig. 14(a). Along the sides, at the centre the field is enhanced by ~ 2 and this increases as we move towards the top edge. Along the top edge, moving from the edge towards the centre, the field decreases rapidly initially and then goes approximately linearly to zero at the centre of the top face. Integration of the current $\boldsymbol{j}$ distribution inside the sample volume (V) gives the moment, m from which the effective susceptibility χ_e can be calculated:

$$m = \frac{1}{2}\int (\boldsymbol{r} \times \boldsymbol{j})dV = -\mu_0 \lambda^{-2} \int (\boldsymbol{r} \times \boldsymbol{A})dV,$$

and

$$\chi_e = \frac{m}{VH},$$

where we have used the linear London equation $\boldsymbol{j} = -\boldsymbol{A}/(\mu_0 \lambda^2)$.

In Supplementary Fig. 14(b) we show the results of performing this calculation for different values of λ. Experimentally, it is useful to calculate the ratio of frequency changes in our resonant coil as λ varies to the change when the sample is completely removed from coil. In the limit that $R \gg \lambda$ this ratio is a linear function of λ and can be expressed as an effective dimension of the sample, $\Delta F_\lambda / \Delta F_0 = \lambda / R_{3D}$. The calculation for our disc gives $R_{3D} = 0.22R$ which compares well to the value estimated in Ref. [25] where in the thin limit $R \gg c$, $R_{3D} = 1/(2+\pi)R = 0.194R$.

To calculate the non-linear response would require solving the non-linear London equations for our geometry which is outside of the scope of the current work. We can however estimate the effect of the field variation across the sample by calculating the volume penetrated by the field into the sample. If we assume a linear response, the penetration is purely exponential and as $\int_0^\infty \exp(-x/\lambda) = \lambda$, we can estimate the volume penetration V_e into the sample from the surface fields, by integrating over the top/bottom and side surfaces. For our disc,

$$V_e = \int_0^c 4\pi R \frac{H(z)}{H_a} \lambda dz + \int_0^R 4\pi r \frac{H(r)}{H_a} \lambda dr. \quad (16)$$

V_e underestimates the difference $m(\lambda) - m(\lambda = 0)$, compared to χ_e by approximately a factor 2 for our disc, because it neglects the changes in the fields outside of the sample. However, it is reasonable to assume we can nevertheless use V_e to estimate the non-linear response by setting λ in Supplementary Eq. 16 to $\lambda(H) = \lambda(0)(1 + \alpha H)$, and then calculate the field dependent enhancement of λ from $V_e(\lambda(H))/V_e(\lambda(0))$. Setting this ratio equal to $1 + \alpha'$, we find that $\alpha'/\alpha = 1.09$, so that the response for this disk is almost the same as the local response (i.e., the response without demagnetising effects). The reason for this is that the effect of the field enhancement on some parts of the sample is cancelled out by the parts close to the centre of the top/bottom faces where the field is lower.

Supplementary Note 10. Estimation of size of $\Delta\lambda(B)$ for LaFePO and $CeCoIn_5$

In general, for a single band d-wave superconductor, H_0 is given by [20]

$$\mu_0 H_0 = \frac{3\mu_\Delta \Delta_0}{2e\lambda_0 v_F} \quad (17)$$

where now, the angular slope of the gap at the node $\mu_\Delta = \frac{1}{\Delta_0}\frac{d\Delta}{d\phi}_{\mathrm{node}}$ is allowed to vary ($\mu_\Delta = 2$ for the d-wave model in Supplementary Eq. 7).

A fit to the linear section of $\Delta\lambda(B)$ (main text Fig. 3) to

$$\Delta\lambda = \alpha B \quad (18)$$

gives $\alpha = 2.1$ nm/mT and 5.1 nm/mT for LaFePO and $CeCoIn_5$ respectively. Note these are the raw numbers without any correction for demagnetising effects. For $CeCoIn_5$ any demagnetising effects are very small because of the $H\|ab$ geometry and for LaFePO ($H\|c$) the demagnetising effects cancel out as explained in the previous section. As $\alpha = \lambda_0/H_0$ and taking $\lambda_0 = 240$ nm for LaFePO [26] and $\lambda_0 = 190$ nm for $CeCoIn_5$ [27] this gives $\mu_0 H_0 = 114$ mT for LaFePO and $\mu_0 H_0 = 36$ mT for $CeCoIn_5$.

Density function theory calculations of the electronic structure of LaFePO show that there is considerable variation in v_F on the different sheets and within each sheet of Fermi surface. The location of the node(s) is presently unknown. v_F ranges from 60 km/s to 500 km/s with an average value of 280 km/s (calculated from the DFT calculations described in Ref. [28, 29]). de Haas van-Alphen measurements [28, 29] show the effective mass is enhanced by a factor 2 compared to DFT and so v_F is reduced by this factor. Assuming $\mu_\Delta = 2$ and $\Delta_0 = 1.43T_c$ (values for single band d-wave), we calculate $\mu_0 H_0$ ranging from 37 mT to 308 mT using Supplementary Eq. (17). For $CeCoIn_5$, the orbitally averaged v_F derived from de Haas-van Alphen effect data [30] ranges from 11 km/s to 50 km/s. With $T_c = 2.1$ K and again assuming a single band d-wave gap, this gives $\mu_0 H_0$ in the range 80 to 370 mT.

Anisotropy in v_F can substantially affect H_0 as the non-linear energy shift depends on the value of v_F at the node, but λ_0 is a weighted average over the whole Fermi surface [20]. In materials, such as $CeCoIn_5$, where there are very strong mass renormalisations, there are additional complications regarding how these renormalisations affect the temperature dependent superconducting properties [31]. Although the above estimated H_0 values are in the same range as the experimental ones, further theoretical work on multiband and strongly interacting superconductors is required before quantitative conclusions can be drawn. In particular, the location of the nodes needs to be known.

---

[1] C. J. S. Truncik, W. A. Huttema, P. J. Turner, S. Özcan, N. C. Murphy, P. R. Carrière, E. Thewalt, K. J. Morse, A. J. Koenig, J. L. Sarrao, et al., Nature Comm. **4**, 2477 (2013).

[2] A. Anderson and R. Peterson, Cryogenics **10**, 430 (1970).

[3] L. Howald, A. Maisuradze, P. D. de Réotier, A. Yaouanc, C. Baines, G. Lapertot, K. Mony, J.-P. Brison, and H. Keller, Physical Review Letters **110**, 017005 (2013).

[4] K. Cho, M. Kończykowski, S. Teknowijoyo, M. A. Tanatar, Y. Liu, T. A. Lograsso, W. E. Straszheim, V. Mishra, S. Maiti, P. J. Hirschfeld, et al., Science Advances **2**, e1600807 (2016).

[5] H. Kawano-Furukawa, L. DeBeer-Schmitt, H. Kikuchi, A. S. Cameron, A. T. Holmes, R. W. Heslop, E. M. Forgan, J. S. White, K. Kihou, C. H. Lee, et al., Physical Review B **88**, 134524 (2013).

[6] C. Putzke, P. Walmsley, J. D. Fletcher, L. Malone, D. Vignolles, C. Proust, S. Badoux, P. See, H. E. Beere, D. A. Ritchie, et al., Nat. Commun. **5**, 5679 (2014).

[7] E. H. Brandt, Phys. Rev. B **60**, 11939 (1999).

[8] S. Majumdar, M. R. Lees, G. Balakrishnan, and D. M. Paul, Physical Review B **68** (2003).

[9] J. R. Cooper, Phys. Rev. B **54**, R3753 (1996).

[10] A. Serafin, A. I. Coldea, A. Y. Ganin, M. J. Rosseinsky, K. Prassides, D. Vignolles, and A. Carrington, Phys. Rev. B **82**, 104514 (2010).

[11] C.-R. Hu, Physical review letters **72**, 1526 (1994).

[12] M. Fogelström, D. Rainer, and J. A. Sauls, Physical review letters **79**, 281 (1997).

[13] Y. S. Barash, M. Kalenkov, and J. Kurkijärvi, Physical Review B **62**, 6665 (2000).

[14] H. Walter, W. Prusseit, R. Semerad, H. Kinder, W. Assmann, H. Huber, H. Burkhardt, D. Rainer, and J. A. Sauls, Physical Review Letters **80**, 3598 (1998).

[15] A. Carrington, F. Manzano, R. Prozorov, R. W. Giannetta, N. Kameda, and T. Tamegai, Phys. Rev. Lett. **86**, 1074 (2001).

[16] M. Araújo and P. Sacramento, Physical Review B **79**, 174529 (2009).

[17] W.-M. Huang and H.-H. Lin, Physical Review B **81**, 052504 (2010).

[18] J. Fletcher, A. Serafin, L. Malone, J. Analytis, J.-H. Chu, A. Erickson, I. Fisher, and A. Carrington, Phys. Rev. Lett. **102**, 147001 (2009).

[19] B. P. Stojković and O. T. Valls, Phys. Rev. B. **51**, 6049 (1995).

[20] D. Xu, S. K. Yip, and J. A. Sauls, Phys. Rev. B **51**, 16233 (1995).

[21] D. Deepwell, D. C. Peets, C. J. S. Truncik, N. C. Murphy, M. P. Kennett, W. A. Huttema, R. Liang, D. A. Bonn, W. N. Hardy, and D. M. Broun, Phys. Rev. B **88**, 214509 (2013).

[22] I. Kosztin and A. J. Leggett, Phys. Rev. Lett. **79**, 135 (1997).

[23] M.-R. Li, P. J. Hirschfeld, and P. Wölfle, Phys. Rev. Lett. **81**, 5640 (1998).

[24] E. E. Chia, D. Van Harlingen, M. Salamon, B. D. Yanoff, I. Bonalde, and J. Sarrao, Phys. Rev. B **67**, 014527 (2003).

[25] R. Prozorov and R. W. Giannetta, Supercond. Sci. Tech. **19**, R41 (2006), ISSN 0953-2048, 1361-6668.

[26] Y. Uemura, Physica B **404**, 3195 (2009).

[27] R. J. Ormeno, A. Sibley, C. E. Gough, S. Sebastian, and I. R. Fisher, Phys. Rev. Lett. **88** (2002).

[28] A. I. Coldea, J. D. Fletcher, A. Carrington, J. G. Analytis, A. F. Bangura, J.-H. Chu, A. S. Erickson, I. R. Fisher, N. E. Hussey, and R. D. McDonald, Phys. Rev. Lett. **101**, 216402 (2008).

[29] A. Carrington, A. Coldea, J. Fletcher, N. Hussey, C. Andrew, A. Bangura, J. Analytis, J.-H. Chu, A. Erickson, I. Fisher, et al., Physica C **469**, 459 (2009).
[30] R. Settai, H. Shishido, S. Ikeda, Y. Murakawa, M. Nakashima, D. Aoki, Y. Haga, H. Harima, and Y. Onuki, J. Phys. Cond. Matt. **13**, L627 (2001).
[31] K. Miyake and C. M. Varma, Phys. Rev. B **98**, 174501 (2018).
